# Mondor’s disease – a rare cause of chest pain: a case report

**DOI:** 10.1186/s13256-017-1530-x

**Published:** 2018-01-09

**Authors:** Navaneethakrishnan Suganthan, Vithiya Ratnasamy

**Affiliations:** 1 Department of Medicine, Teaching Hospital, Jaffna, Sri Lanka; 2 Professorial Medical Unit, Teaching Hospital, Jaffna, Sri Lanka

**Keywords:** Chest pain, Mondor’s disease, Thrombophlebitis

## Abstract

**Background:**

Chest pain is one of the common presenting symptoms encountered in an emergency department. Prompt history taking and careful clinical examination do help to differentiate cardiac chest pain from other causes. Mondor’s disease is a rare cause of chest pain which is often underdiagnosed due to lack of awareness. Mondor’s disease is a condition characterized by thrombophlebitis of the superficial veins of breast and anterior chest wall. The diagnosis is often made clinically.

**Case presentation:**

Here we report a case of a 37-year-old Sri Lankan Tamil woman who presented with chest pain and was clinically diagnosed as having Mondor’s disease after a physical examination, which was confirmed with demonstration of thrombophlebitis by ultrasound scan imaging. Although it is a self-limiting condition, non-steroidal anti-inflammatory drugs are used in the treatment to hasten recovery in addition to giving reassurance.

**Conclusions:**

Mondor’s disease is not considered a differential diagnosis for chest pain due to lack of awareness of this medical condition. Creating awareness of this condition via this case would help to cut down unnecessary investigations and valuable time spent in emergency departments, and it helps to identify a serious underlying cause especially carcinoma of the breast at its early stage.

## Background

Chest pain is a common presentation encountered in clinical practice. Mondor’s disease is a rare cause of chest pain which is often underdiagnosed due to lack of awareness. Mondor’s disease is a condition characterized by thrombophlebitis of the superficial veins of the breast and anterior chest wall. Initially this condition presents with rapid development of a subcutaneous red cord-like lesion that causes pain at an early stage and subsequently becomes a painless fibrous band. The diagnosis is often made clinically. Demonstration of thrombophlebitis by ultrasound scan imaging further supports the diagnosis. Although it is a self-limiting condition, non-steroidal anti-inflammatory drugs are used in the treatment to hasten recovery. Here we report a case of a 37-year-old Sri Lankan Tamil woman who presented with chest pain and was diagnosed as having Mondor’s disease.

## Case presentation

A 37-year-old Sri Lankan Tamil woman presented to the emergency department of our tertiary care center with a history of left-sided chest pain of 1-week duration. The pain was not related to excretion or exercise and she had no symptoms suggestive of pleurisy. She could not recall any trauma to her chest and denied mastalgia or discharge from nipple. She had no systemic symptoms such as fever, loss of appetite, or loss of weight. Further, she did admit that she had been extensively investigated by her general practitioner including electrocardiogram (ECG) and two-dimensional echocardiogram (ECHO) to identify the cause of her chest pain without success prior to this admission. She had no significant past medical or surgical history of note. She had no risk factors for ischemic heart disease and none of her first-degree relatives were diagnosed as having either breast carcinoma or thrombophilia. She has regular menstrual cycles with normal flow and she is not practicing any contraceptive methods. She is a housewife, looking after two children.

**Fig. 1:**
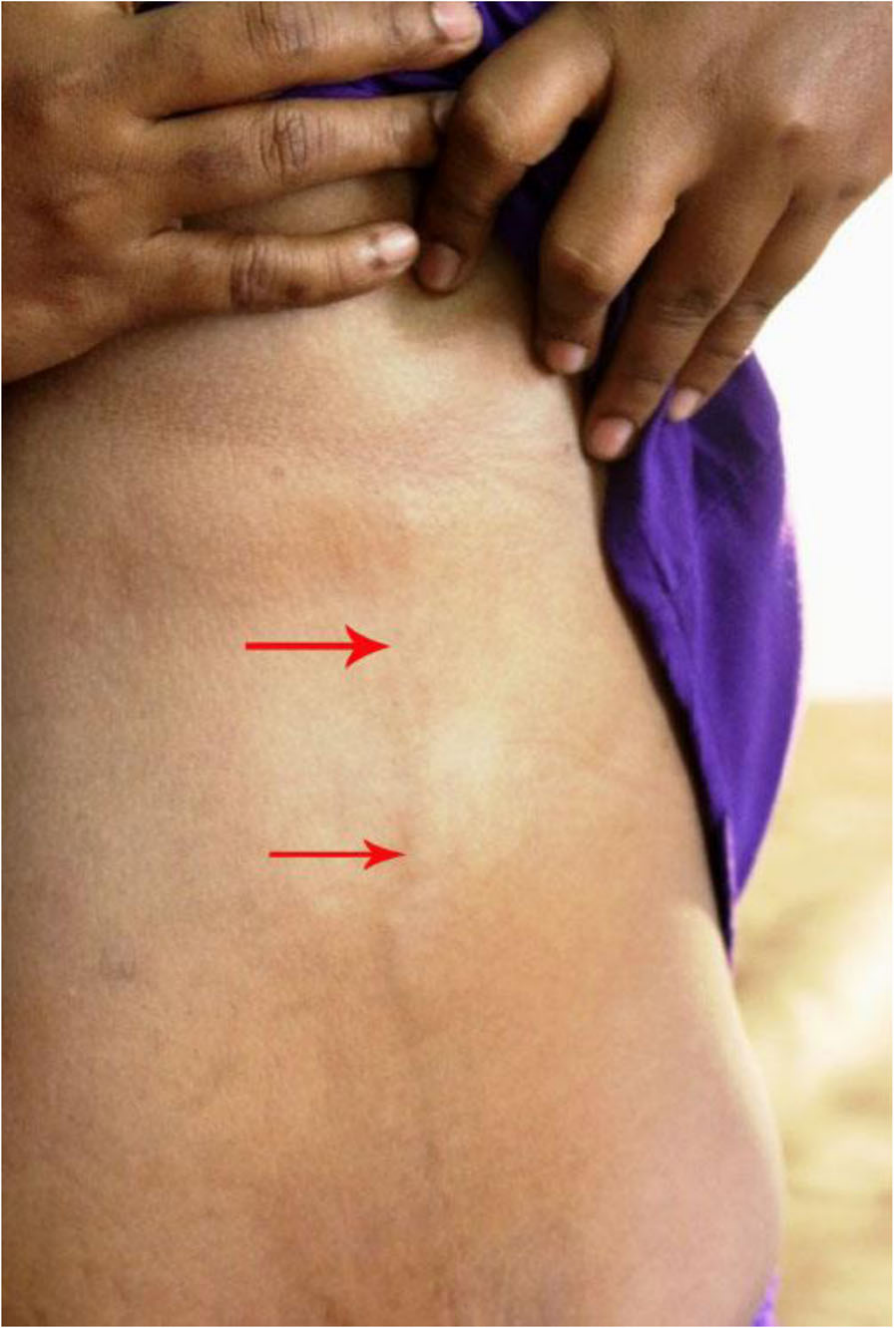
Palpable cord-like structure (Left thorocoepigastric vein) made prominent by elevation of breast tissue (*red arrow*).

An examination revealed a tender area on left anterolateral part of her lower chest and abdomen with a vertical cord-like swelling just under the skin extending from lower part of her left breast to iliac fossa which was made prominent by abduction of her left arm and elevation of her left breast (Fig. 1); these finding were compatible with a clinical diagnosis of inflammation and thrombosis of left thoracoepigastric vein. No abnormalities were found on examination of her breast and there was no regional lymphadenopathy. She had no physical signs suggestive of vasculitis. The rest of the clinical examination was unremarkable and all vital signs were within normal range.

**Fig. 2:**
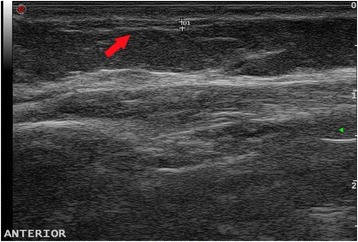
Ultrasound scan showing dilated superficial vein (Left thorocoepigastric vein) with anechoic lumen (*red arrow*)

Based on the history and clinical examination a clinical diagnosis of superficial thrombophlebitis (Mondor’s disease) was made. An initial laboratory work-up including full blood count, erythrocyte sedimentation rate, renal profile, and liver profile was normal. An ECG showed no abnormalities. Gray-scale ultrasonography showed non-compressible left thoracoepigastric vein containing hypoechoic material in the subcutaneous fat with multiple areas of narrowing giving beaded appearance (Fig. 2) and color Doppler revealed no flow signal which was suggestive of thrombophlebitis with thrombosis. She had no deep vein thrombosis and an ultrasound scan of both breasts and axilla revealed no abnormalities. She was reassured and treated with a course of ibuprofen and her symptoms resolved completely at 4 weeks. Subsequently she was lost to follow up.

## Discussion

Mondor’s disease, as originally described by Mondor in 1939, is a superficial thrombophlebitis of anterior chest wall [1]. In 1958, Helm and Hodge reported a case of thrombosis of dorsal superficial vein of penis as penile Mondor’s disease [2]. Thoracoepigastric, superficial epigastric, and lateral thoracic veins are the most common sites of involvement. In our patient, the thoracoepigastric vein was involved. Although hormone therapy, breast cancer, thrombophilic conditions, and surgical or physical trauma, have been identified as the common etiological factors, mostly commonly it is idiopathic [3]. It is three times more common in females than in males [4].

Based on the history, clinical examination, and investigations, no risk factor was identified in this patient. Patients usually present with sudden onset of chest pain and a palpable tender erythematous cord-like structure is found corresponding to the anatomical location of the superficial veins of the chest wall or breast [5].

Even though the pathophysiology of this condition is not very clear, it is postulated that direct trauma, pressure on the vein with stagnation of blood, and stretching and relaxing of the vein are the underlying mechanisms for development of Mondor’s disease [6]. In an ultrasound scan, the thrombosed vessel appears as a superficially located, long, tubular, anechoic structure with a beaded appearance and absent color flow in Doppler studies [7]. In patients with palpable findings in the breast examination, mammographic examination is essential to rule out an underlying malignancy [7].

Although it is a self-limiting condition, local application of heat and topically or orally administered non-steroidal anti-inflammatory drugs can be used to alleviate the symptoms. Antibiotics are not necessary unless there is evidence of infection [8, 9]. Systemic anticoagulation with low molecular weight heparin or orally administered anticoagulants is recommended only for high-risk patients. Reduction in thrombus size and pain has been observed with locally acting anticoagulants, and it can be used in low-risk patients [10].

Chest pain is a common presentation encountered in clinical practice. Our patient presented with chest pain and was found to have the typical features on examination; diagnosis of Mondor’s disease was made based on the history and examination. No risk factor or underlying cause was identified in this case and diagnosis was confirmed further with duplex scan. Her symptoms resolved in 4 weeks with non-steroidal anti-inflammatory drugs. Mondor’s disease is a rare cause of chest pain which is often underdiagnosed due to lack of awareness. Failure to recognize this mostly benign self-limiting condition can result in unnecessary investigations and cause significant anxiety to the patient.

## Conclusions

Even though Mondor’s disease is an uncommon cause of chest pain, it is not considered a differential diagnosis due to lack of awareness of this condition. At the same time it can be a rare manifestation of an underlying serious condition especially breast carcinoma. It is easy to diagnose at the bedside by performing a clinical examination. Creating awareness of this condition via this case would help to cut down costs and valuable time in emergency departments by avoiding unnecessary investigations.
